# Theorising pathways of multi-dimensional scaling for population health using implementation and complexity science perspectives

**DOI:** 10.1186/s43058-026-01021-x

**Published:** 2026-07-07

**Authors:** Monika Martens, Edwin Wouters, Josefien van Olmen

**Affiliations:** 1 Faculty of Medicine, Department of Family Medicine and Population Health (FAMPOP), University of Antwerp, Antwerp, Belgium; 2 Faculty of Social Sciences, Department of Sociology, University of Antwerp, Antwerp, Belgium; 3 Centre for Research in Health System Performance (CRiHSP), Yong Loo Lin School of Medicine, Department of Family Medicine (DFM) National University Health System, National University of Singapore, Singapore, Singapore

## Abstract

Scaling up is a complex process. As a multi-dimensional concept, it requires efforts to (1) increase population coverage (coverage), (2) expand or diversify what is included in the health service package (expansion), and/or (3) institutionalise a health innovation or new practice into health system services (institutionalisation). In this paper, we provide the theoretical basis for the model joining linear as well as complex pathways – stemming from implementation and complexity science – towards three-dimensional scaling. Our scale-up model positions expansion as the backbone for scale-up, and proposes multiple back-and-forth waves between institutionalisation and coverage. This allows an incrementalist approach, going step-by-step from one to the other scale-up dimension, as well as a multi-player complexity approach, emphasising the interactions between scale-up dimensions and actors involved, to achieving population health. By offering a dual incrementalist-complexity focus, we acknowledge that there is a starting point to scale-up, and thus path dependency, in addition to highly contextual cultural, historical, socio-political, and economic forces that underpin population health and any attempt at scaling up access and integration of health services, thereby pointing at their fragmented and incomplete nature. The visualisation and underlying hypotheses offer speculation on potential pathways for scale-up, which are key processes to understand and untangle in future research.

## Studying scale-up for population health: why and how?

The world demonstrated during the COVID-19 pandemic that large-scale public health action can be mobilised with remarkable speed. Testing, social distancing, masks, and vaccines were scaled up in many parts of the world in a matter of months. Why then, decades into the evidence base, do we continue to stumble in scaling equally vital population health interventions—ensuring quality education, clean water and sanitation, essential medicines and health services, including both wider and targeted prevention and promotion, such as the WHO “Best Buys” for noncommunicable diseases? Why does improving air quality, housing, urban design, and equity still feel perpetually “out of reach”? The answer lies less in the absence of solutions than in the politics and practice of scale-up. Conflicting policy agendas and entrenched interests constrain progress [1], while the complexity of scaling up itself leaves many policymakers and implementers adrift, caught between evidence and action [2, 3]. With the political landscape remaining largely beyond our control—though demanding sustained advocacy, communication and capacity-building efforts—, it is our task as implementation scientists, to unravel the inherent complexity of scaling interventions to move scale-up practice and science forward [4, 5].

In population health, scale–up pertains to ‘those efforts that increase the impact of health interventions for the benefit of more people, whilst fostering sustainable policies and programmes’ [6]. This WHO definition is underpinned by clear population health principles: to scale *equitably, sustainably, efficiently* and *effectively*. The fact that these goals can be linked to somewhat contrasting ideologies adds to the complexity of scale-up practice.

To support scale-up practice, scale-up science is emerging [7–11]. So how can we further advance it? Aside from the practical scale-up knowledge that is available through projects and generic frameworks [6, 8, 10, 12, 13], theory is indispensable for planning, guiding, monitoring and evaluating scale-up [9, 14–17]. Yet, it is not only theories as finished products that matter [18]. As Kislov argues, the act of *theorising* itself—treating theory as a dynamic process—creates space for genuine dialogue between data and ideas, driving conceptual and practical innovation [18]. This shift invites us to move beyond passive application of frameworks toward active engagement with theory [19, 20], with the potential to transform how we understand, design, and evaluate scale-up [2, 16, 17].

For this, there needs to be a nuanced understanding of different scale-up perspectives or paradigms [9]. As can be expected from any ambiguous and contested arena, in which technicalities are hard to separate from politics [21–23], different conceptualisations and perspectives of scale-up co-exist [8, 24, 25]. The study of scale-up spans a spectrum from implementation research perspectives with a predominantly more linear view on scale-up, towards complexity perspectives that consider scale-up as adaptive change in a system emphasizing the unpredictability, self-organisation, and interdependencies of that system [2, 9]. While complexity science increasingly informs scale-up science [11, 26], they remain underutilised in scaling up public health interventions [2]. Implementation science approaches have dominated the scale-up literature; consequently, public health scale-up models have focused on replicating interventions through linear spread [2, 4, 12].

This paper provides a new theoretical insight on scale-up by combining these perspectives. Through theorising and theory layering, we expand upon the conceptualisation of scale-up used in the SCUBY project [27] based upon WHO/ExpandNet strategies [6], by connecting it with the existing prevailing perspectives on scale-up. We present a theorising exercise that aims to generate hypotheses. As such, this commentary has a limited empirical base and is therefore primarily a theoretical endeavour, drawing on meta-level insights from the SCUBY project [28] and an integrative analytical reflection conducted as part of the first author’s doctoral research [29]. The underlying insights and the theory layering process are described in the sections that follow.

## SCUBY’s scale-up experience and conceptualisation

**Fig. 1:**
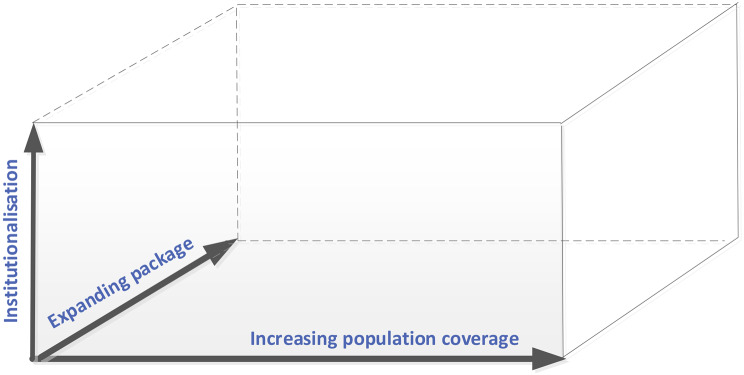
Three-dimensional scale-up framework (adapted from van Olmen et al. [27], with strategies based upon WHO/ExpandNet [6] and three-dimensional framing based upon Meessen et al. [32], inspired by the universal health coverage framework [33])

In the ‘SCale-Up of integrated care for Diabetes and hYpertension’ (SCUBY) project, a European-funded project which ran from 2019 to 2023, we facilitated and evaluated the scale-up of integrated care for these chronic conditions via the organisation of policy dialogues and the development and (partial) implementation of evidence-informed scale-up roadmaps in Belgium, Cambodia and Slovenia [27]. These two implementation strategies – policy dialogues as a collaborative governance instrument and roadmaps as a strategic planning and coordination tool – were pragmatic steps we discerned in the pathway towards scale-up [29, 30]. The intricate relations between these strategies and their role in scale-up are described elsewhere [31]. In SCUBY, scale-up was broadly defined, leaving ample room for both linear and complex pathways to scale-up. We conceptualised scale-up as an approach consisting of efforts to (1) increase population coverage, (2) expand or diversify what is included in the integrated care service package, and/or (3) institutionalise integrated care into health system services [27, 32, 33]. Figure 1 showcases the three-dimensional scale-up framework. Each dimension can be related to underlying population health principles (cf. overarching health system goals) of equity (cf. coverage), sustainability (cf. institutionalisation), efficiency and effectiveness (necessitating quality improvement projects and health innovations and thus expansion) (see appendix 1 for glossary of terms and appendix 2 for more detailed description and definitions). These dimensions further align with WHO’s terminology of horizontal and vertical scaling strategies [6], corresponding to coverage and institutionalisation, respectively (cf. appendix 2). The framework presented in Fig. 1 adds expansion as a third dimension, which further corresponds to WHO’s diversification strategy [6].

The lessons learned in SCUBY from the three empirical country case-studies on scaling up integrated care in vastly different health systems [34] and its resulting theoretical reflection led to the metaphor of a spiral process [28]. The spiral metaphor fits to show the interactions and levels of scale-up dimensions: where (i) an enabling and elastic environment is a pre-requisite for expanding the package of care and vice versa, expanding a package of care can be part of creating an enabling environment and thus health system strengthening (operational integration) efforts, (ii) subsequent dialogue in collaborative governance and policy-making efforts is required to institutionalise integrated care within existing governance structures (system integration), to (iii) then adopt diversification strategies that focus on specifically vulnerable populations to not be left behind (equitable coverage through proportional universalism) [28]. Those overarching strategies we found, already pointed at a certain ‘order’ or incrementalism to scaling, ranging from health system strengthening, to collaborative governing and policy-making and health equity promotion, and also align with the population health principles of effectiveness and efficiency; sustainability; and coverage, respectively.

## Exploring perspectives on scale-up

Koorts [2] introduces a new perspective on scaling that shifts the focus from the intervention itself to achieving desired population-level health outcomes and proposes that ‘scaling up’ should be seen as existing on a continuum:

- at one end, effective scaling may involve a linear, intervention-focused approach that emphasizes *spreading evidence-based interventions ‘into’ existing systems* to expand their application. This is the dominant, traditional model and its focus is on the interaction between an intervention’s attributes and external contexts, giving rise to concepts such as intervention plasticity and context elasticity or ‘innovation-system fit’, which is a critical feature of a system’s readiness and preparedness to accept change brought about by an intervention [16];
- at the other end, scale-up can be viewed through a complex systems lens, where interventions are understood as events within broader systems. Implementation and scale-up efforts should aim to *generate systemic changes ‘within’ the system* to achieve the desired outcomes. Koorts refers to this as ‘systems-oriented scale-up’ for improving population health, which can complement traditional approaches in suitable situations.

## Proposing archetypes and pathways of scale-up

For the sake of enriching these paradigms via ‘theory layering’, we argue that it would be relevant to apply this approach—the intervention to systems-oriented scaling continuum—to the various dimensions of scale-up to reflect on the repercussions for each. By combining the continuum of scale-up perspectives of Koorts with SCUBY’s three-dimensional scale-up framework, we came to six different scale-up archetypes (see Table 1), that can be part of a scale-up pathway. Elaborated examples across the six scaling archetypes can be found in appendix 3.

**Table 1:**
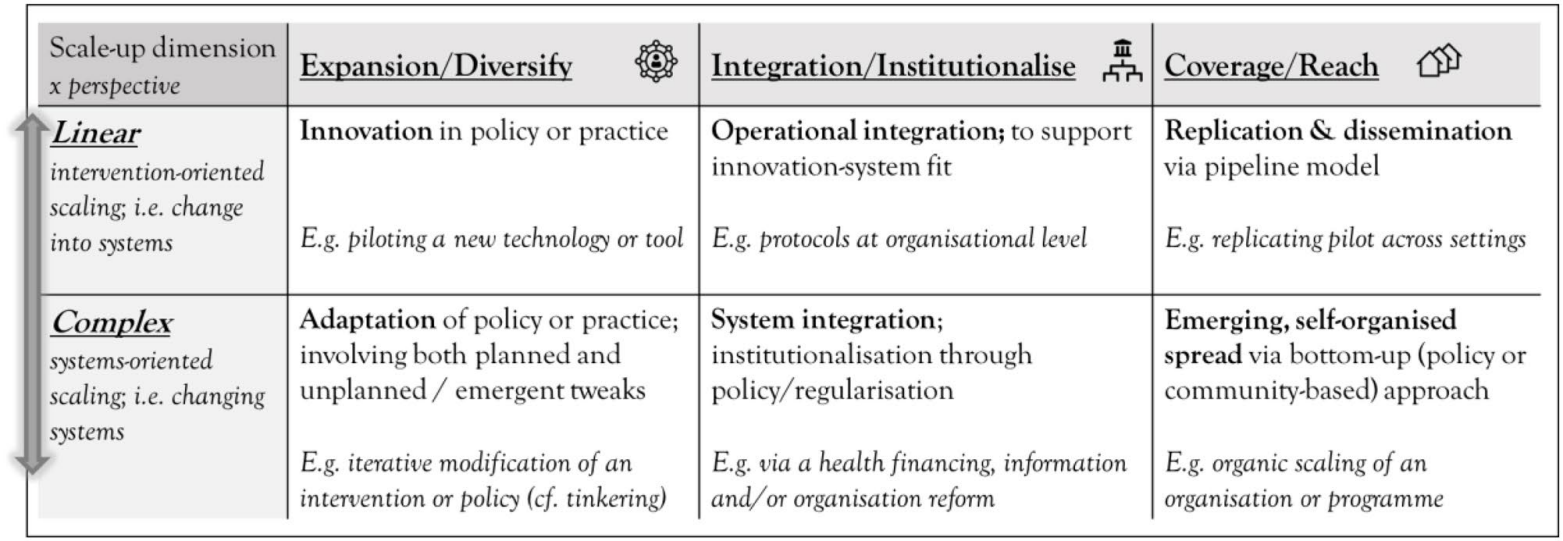
Exploring scale-up archetypes

**Fig. 2:**
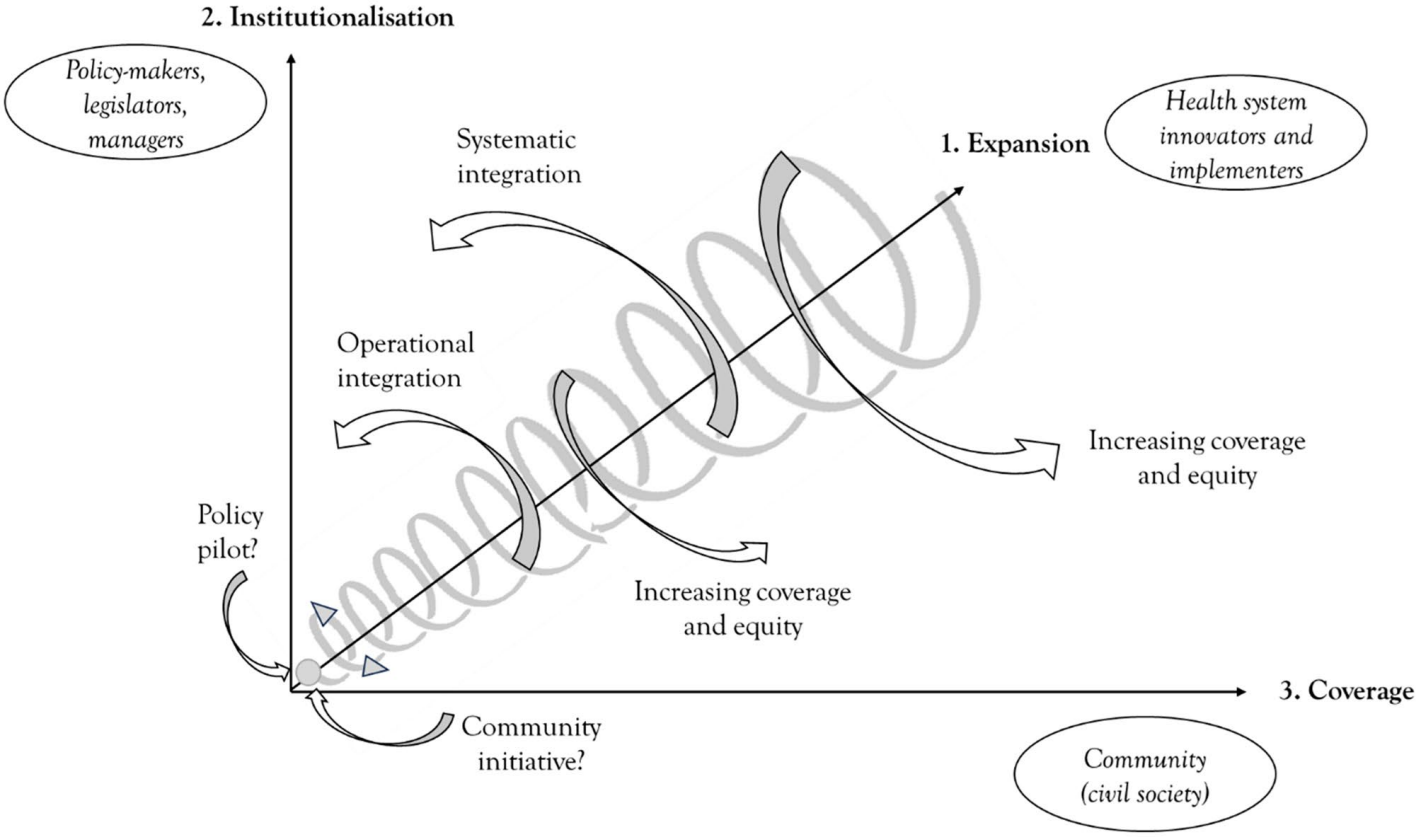
A revised scale-up spiral model for simultaneous theorising on order (from linearity and incrementalism perspective) and on interacting relations between scale-up dimensions (from complexity perspective)

Next to the practical tools and strategies that can ‘guide’ a scale-up pathway in practice, the six scale-up archetypes exist as theoretical ‘typologies’ and, –as practice shows – are often used in combination and thus can make up a scale-up pathway, i.e. scaling by: 1) innovation; 2) adaptation; 3) operational integration; 4) institutional integration; 5) replication & dissemination; and 6) emerging, self-organised spread. Table 1 thereby offers different ways of viewing not just systems, but also agency. The linear intervention-oriented scale-up approach showcases scaling as efforts mainly from a single/external player perspective (e.g., researcher, innovator, or manager); while the complex systems-oriented approach to scaling considers a more holistic, multi-player perspective to scaling, envisioning the concertation of diverse stakeholders and stakeholder groups (e.g., end-users, practitioners, managers, policy makers, interest groups) as a requirement for success.

Koorts [2] argues that traditional intervention-oriented approaches remain highly appropriate in many circumstances and that complex systems approaches are by no means universally required for scale-up. Scaled-up interventions adopt many different pathways [35] and a complex problem does not always require a complex systems approach when scaling. Combinations are perfectly possible, e.g., “a population intervention can be embedded in a national strategy [i.e. policy adaptation] that adopts a systems perspective [i.e. aimed at operation or systems integration], and yet the strategy used to plan and undertake national roll-out of that intervention can remain linear [coverage via replication]” [2]. This highlights how various scale-up dimensions may be involved and working together, as a pathway.

The question that arises from Table 1 is whether the *expansion* component can be considered a fully-fledged scale-up dimension or remains within the realm of implementation. We argue that it offers a pathway to scaling, as it entails the intent for larger scale and that expansion as scale-up in itself, would not occur without institutionalisation or coverage. This is not to say that expansion is less relevant to scale-up science, or as a dimension, as mostly scale-up efforts—depending on the current policy stage—start with a ‘seed’, a blueprint, an innovation or desire for system change/adaptation.

The six scale-up archetypes thus allow critical reflection on what constitutes scaling and how to distinguish between implementation and scale-up. As partially overlapping concepts, there are two possible views on this: (a) implementation can be viewed as preceding scale-up [36] (no scale-up without implementation first), or (b) scaling involves implementation in itself [4]. We argue that scale-up can follow pilot (implementation), though implementation does not necessarily need to precede scale-up. System-wide roll-out of initiatives or system integration through institutional reform can follow straight from policy design and adoption. Or scale-up can emerge, bottom-up, self-organise, and/or follow from operational integration efforts.

### Hypotheses on how the scale-up dimensions converge (or not) and spiral

Building further on the distinction made between single-player-led scaling and multi-player-led scaling and scale-up archetypes, we propose several hypotheses about the interdependency and timing within scale-up pathways, visualised in Fig. 2. The first two hypotheses regard the linear pathway to scaling:

1. From a single-player perspective, if no co-production is set up or emerges, the first ‘step’ to scaling could be taken by either a policy or a health system/community actor^1^. Whereas increasing *coverage* in a linear way involves an equity-oriented approach to ‘expand’ and introduce a population health intervention or health system change through replication and dissemination either for the broad population or specific communities and at-risk groups and whereas *institutionalisation* primarily involves policy and managers, *expansion*—associated with health service effectiveness and efficiency—supports health system strengthening in a more direct fashion than policy and institutionalisation efforts allow. Therefore, a logical deduction would be to start close to the health system and *expand* innovations, in response to feedback from policy and (professional and civil society) community actors. In other words, the first step to scaling is to start small, to test and experiment, monitor and evaluate.
2. As a second step, policy actors and managers would enable this innovation through *institutionalising* it—entailing both operational and systematic integration. And thirdly, communities as well as health system and policy actors would call for equity and horizontal scale-up, the *coverage* dimension. The reason why ‘integration/institutionalisation’ precedes ‘coverage’ is that even with scaling from (grassroot) communities, some type of protocol, guide, business model, incentive, standard operating procedure, blueprint, or proof of concept would precede community bottom-up scaling efforts (cf. operational integration). From a technical point of view, this logic may explain why equity often remains an afterthought, with as example the global COVID-19 vaccination roll-out revealing a deficiency in equitable access mechanisms due to a myriad of proprietary legal aspects and procedures, resulting in a significant gap in vaccine coverage between high- and low-income countries. From the political side even more so, this proposition can be explained by ‘the politics of scale-up’ shaped by political priorities and neoliberal ideologies that favour efficiency and effectiveness over equity.

The third, core hypothesis is related to the complexity perspective and the dual incrementalist-complexity focus possibility:

3. From a multi-player complexity perspective, scale-up emerges or becomes an option as a result of the tension between both policy and health system/community actors^1^, possibly through co-production and concertation or advocacy efforts. Figure 2 with a revised spiral model showcases this tension as well as the theorised ‘order’ simultaneously, whereas both perspectives may be useful in unravelling scale-up pathways. With expansion as its benchmark/core, we hypothesise that scale-up moves in waves, back and forth between institutionalisation and coverage. Expansion itself can start from a policy pilot with institutionalisation intent or a community initiative from a coverage and equity perspective. This shows how the dimensions are rooted in or around expansion and how this proposition for scale-up conceptualisation offers an incrementalist approach to achieving population health, which is not opposite from a complexity perspective. Rather, it recognises how existing practices can be shaped and reshaped through both small emergent tweaks and tinkering as well as complex big-scale reform. After all, the incremental/expansion benchmark is in constant interaction with the other scale-up dimensions. That said, this scale-up conceptualisation not only approaches incrementalism, but offers a dual incrementalist-complexity focus, acknowledging that there is a starting point, and thus path dependency, in addition to highly contextual cultural, historical, socio-political, and economic forces that underpin population health and any attempt at scaling up access and integration of health services, thereby pointing at their fragmented and incomplete nature.

The application of the three-dimensional scaling framework may have different implications for high-income countries (HICs) and low- and middle-income countries (LMICs), particularly regarding the role of donor funding as a structural determinant of institutionalisation. In many LMIC contexts, donor funding promotes continued expansion through the addition of interventions by different external actors, often reinforcing fragmented “pilot-itis” dynamics. Some initiatives move towards greater coverage through collaboration with the public sector, as illustrated by programmes such as the World Health Organization Package of Essential Noncommunicable (PEN) disease interventions for primary health care [34]. However, institutionalisation often remains limited because donor dependency itself contributes to temporality, fragmentation, selective geographical or population targeting, and insufficient structural support for health system constraints such as workforce and financing shortages. By contrast, in HICs, scaling tends to cycle through recurring waves of innovation and adaptation—such as digital health, artificial intelligence, or peer-support initiatives—yet faces its own persistent barriers: horizontal barriers to coverage, alongside vertical barriers to institutionalisation, including unresolved or emerging legal and regulatory challenges [28, 34].

## Conclusion

In this paper, a three-dimensional scale-up framework [27] developed in the SCUBY project, aligning with the three key underlying population health principles of equity, sustainability and effectiveness and efficiency was combined with Koorts’ [2] continuum of linear-complex scaling approaches, in a theorising process of theory layering. The revised scale-up spiral model uses the expansion scale-up dimension as its backbone and proposes that scale-up moves in waves, back and forth between institutionalisation and coverage. The structuring of scale-up efforts can aid the discovery of pathways of policy and programme change towards equity, sustainability and effectiveness. Our newly proposed theory on scale-up pathways – accompanied by a scale-up spiral visualisation and three underlying hypotheses – offers speculation on potential pathways for scale-up, which are key processes to understand and untangle in future research. We thus call on scale-up scientists to use, test, and refine this new theory on scale-up.

## Data Availability

No datasets were generated or analysed during the current study.

## Appendices

### Appendix 1: glossary of terms used

*Scale-up dimensions:* Coverage, expansion and institutionalisation (definitions in appendix 2)

*Population health principles (and health system goals):* Equity, sustainability, efficiency and effectiveness (cf. appendix 2 under ‘aim’)

*Scale-up continuum:* From linear implementation to complex scaling perspectives/approaches

*Scale-up strategies:* Horizontal, diversification and vertical scaling (alternatively, specific actions taken to scale-up; e.g. organising policy dialogues and developing roadmaps)

*Scale-up pathways:* How the spiral moves in between scale-up dimensions

*Scale-up archetypes:* The three scale-up dimensions can be mapped onto the linear to complex scale-up continuum and results into a 3 × 2 matrix with following six archetypes: a) expansion as innovation (1) or adaptation (2); b) institutionalisation as operational integration (3) or system integration (4); c) coverage as linear replication/dissemination (5) and as emerging, self-organised spread (6).

### Appendix 2: Definitions, synonyms, perspectives, aims and metaphors of the three scale-up dimensions

**Table:**

Scale-up dimensions	Coverage (cf. reach)	Expansion (cf. diversification)	Institutionalisation (cf. systems integration)
**Definition from WHO/Expandnet, 2010** [6]	*Horizontal* scale-up refers to the expansion and replication of an innovation. Innovations may be replicated in different geographic sites or can be extended to serve larger or different population groups	*Diversification,* also called functional scaling up or grafting, consists of testing and adding a new innovation to one that is in the process of being scaled up. It explores the possibility of pilot testing an added component to the innovation	*Vertical scale-up* refers to the policy, political, legal, regulatory, budgetary or other health systems changes needed to institutionalize the innovation at the national or sub-national level
**Definition from van Olmen, 2020** [27]	The population coverage, measured by reach, and by number of people actually covered by intervention	The expansion of the intervention/benefit package towards the ICP, measured through ICP implementation assessment	Integration into health system and services; the extent to which complex systems (structure and processes) allow (maintain and institutionalise) ICP implementation
**Synonyms**	Reach [37]; spread, expansion, replication [9] Scaling ‘out’ [7]	Diversification [6], cf. piloting an innovation, adaptation or modification Scaling ‘deep’ [7]	Maintenance [37]; penetration, sustainability [38] Scaling ‘up’ [7]
**Perspective**	Implementer ‘From below’	Implementer ‘From below’	Planner ‘From above’
**Aim**	Equity [39]; magnitude of impact [7] (i.e. population health improvement)	Improving quality and/or comprehensiveness of service package [7] and thus efficiency and effectiveness	Sustainability of impact via systems strengthening [2, 24, 25]
**Metaphor of the apple tree** [7]	From one tree, to many trees and more fruit: Scaling *out* expands sites or opportunities (increasing scale requires more inputs + generates more outputs).	From one tree, to same (size) tree, with enhanced fruit by locally fertilising (branches): Scaling *deep* affects quality and character (larger and sweeter apples)	From one tree, to big tree(s) and more fruit, by fertilising the earth: Scaling *up* increases throughput (e.g., more efficient processes following capacity-building and training)
	Aim: to grow more apples	Aim: to improve the quality/variety of the apples	Aim: to grow more apples, in better conditions

*Note: ICP: integrated care package*

### Appendix 3: Elaborated examples across six scaling archetypes

**Table:**

Scale-up dimension *×* perspective	Expansion/Diversify	Integration/Institutionalise	Coverage/Reach
Linear perspective *intervention-oriented scaling; i.e. change into systems*	**Innovation** *E.g. piloting a new technology or tool;* *E.g. adding a new, evidence-based screening service to an existing primary care offer* *E.g. adopting a guideline-recommended practice with fidelity in a new setting*	**Operational integration** *E.g. developing and adopting protocols and guidelines at organisational level* *E.g. standardising operating procedures and referral pathways between primary and secondary care* *E.g. embedding a new intervention into existing care routines (e.g. integrating mental health screening into routine GP consultations)*	**Replication & dissemination** *E.g. replicating a pilot across further units, practices or localities* *E.g. increasing health coverage by training more health professionals* *E.g. expanding population access to a preventive programme across additional geographical areas (e.g. extending vaccination outreach to previously unreached districts)*
Complex perspective *systems-oriented scaling; i.e. changing systems*	**Adaptation** *E.g. a health improvement intervention iteratively modified by frontline practitioners to fit local cultural norms and population needs (e.g. via PDSA cycles)* *E.g. adding strategies or themes to a health policy plan (cf. tinkering) in response to emerging field evidence (e.g. broadening an NCD strategy to incorporate mental health after co-morbidity burden becomes apparent)* *E.g. diversifying the role of community health workers from disease-specific tasks (e.g. TB contact tracing) to broader primary care functions (e.g. chronic disease monitoring and health promotion) (cf. task-shifting)*	**System integration** *E.g. investing in workforce education and training (e.g. embedding health literacy into professional training frameworks)* *E.g. installing Health-in-All-Policies (HIAP) measures via collaborative, multi-sectoral governance* *E.g. health systems strengthening via health financing, information and/or organisation reform (e.g. restructuring financing mechanisms to sustain scaled interventions)*	**Emerging, self-organised spread** *E.g. practitioners supporting each other via informal training/peer support networks* *E.g. community or patient advocacy movement raising public awareness and pressuring local authorities to expand access (e.g. around mental health or environmental health issues)* *E.g. NGO formation and organic scale-up of community-based health programmes across underserved areas*

*Note: Examples are illustrative and intended to be transferable across public health topics. PDSA = “Plan-Do-Study-Act” cycles; Cf. = compare with; NCD = non-communicable disease; GP = general practitioner; HIAP = Health-in-All-Policies; NGO = non-governmental organisation*

## Footnotes

1 The rationale for putting together health system and community actors here is that both coverage and expansion scale-up dimensions are concerned with health system change, while institutionalisation is concerned with (health system change via) health policy change.
